# Amyloid and tau PET in cerebral amyloid angiopathy-related inflammation two case reports and literature review

**DOI:** 10.3389/fneur.2023.1153305

**Published:** 2023-04-28

**Authors:** Jhih-Yong Yang, Yung-Tsai Chu, Hsin-Hsi Tsai, Jiann-Shing Jeng

**Affiliations:** ^1^Department of Neurology, National Taiwan University Hospital, Taipei, Taiwan; ^2^Department of Neurology, National Taiwan University Hospital Bei-Hu Branch, Taipei, Taiwan

**Keywords:** cerebral amyloid angiopathy-related inflammation, magnetic resonance imaging, amyloid positron emission tomography (PET), cerebral amyloid angiopathy (CAA), microbleed

## Abstract

**Background:**

Cerebral amyloid angiopathy-related inflammation (CAA-ri) is a clinical syndrome characterized by MRI findings of amyloid-related imaging abnormalities-edema (ARIA-E) suggestive of autoimmune and inflammatory reaction and hemorrhagic evidence of cerebral amyloid angiopathy. The longitudinal variation of amyloid PET and its imaging association with CAA-ri are undetermined. Moreover, tau PET in CAA-ri has been rarely investigated.

**Method:**

We retrospectively described two cases of CAA-ri. We provided the temporal change of amyloid and tau PET in the first case, and the cross-sectional finding of amyloid and tau PET in the second case. We also performed a literature review of the imaging features of amyloid PET in reported cases of CAA-ri.

**Results:**

In the first case, an 88-year-old male presented with progressive consciousness and gait disturbances over 2 months. MRI showed disseminated cortical superficial siderosis. Amyloid PET prior to and after the CAA-ri revealed focally decreased amyloid load in the region of ARIA-E. In the second case, a 72-year-old male was initially suspected to have central nervous system cryptococcosis but later diagnosed with CAA-ri because of the characteristic MRI features and good response to corticosteroid treatment; a subsequent amyloid scan revealed positive amyloid deposition of the brain. Neither case suggested an association between the region of ARIA-E and higher amyloid uptake on PET before or after onset of CAA-ri. Our literature review revealed variable findings related to amyloid burden in post-inflammatory regions in previously reported CAA-ri cases with available amyloid PET. Our case is the first report of longitudinal changes on amyloid PET and show focal decreases in amyloid load after the inflammatory process.

**Conclusion:**

This case series highlights the need to better explore the potential of longitudinal amyloid PET in the understanding of the mechanisms of CAA-ri.

## Introduction

Cerebral amyloid angiopathy-related inflammation (CAA-ri) is a rare immune-mediated disorder of the central nervous system with histopathologic findings of perivascular inflammation in the setting of cerebral amyloid angiopathy (CAA) (1–4). The clinical presentation of CAA-ri is highly variable and depends on the region of brain inflammation, and can present as cognitive decline, memory impairments, confusion, personality changes, seizures, or an altered level of consciousness (5).

Confirmative diagnosis of CAA-ri requires histopathological evidence. Clinico-radiological diagnostic criteria for CAA-ri were validated by Auriel et al. (6), and require detection of unifocal or multifocal asymmetrical white matter hyperintense lesions that extend to the immediate subcortical white matter in a symptomatic patient with concomitant CAA hemorrhagic lesions on MRI. Radiotracers for amyloid positron emission tomography (PET), such as 11C-Pittsburgh Compound B (PiB), 18F-Florbetapir, or 18F-Florbetaben, may have additional value for the clinical diagnosis of CAA (7–10) and CAA-ri (11–13). However, few studies have applied amyloid PET specifically for CAA-ri, especially to investigate the temporal changes on follow-up imaging. Here, we describe the clinico-radiological presentations and MRI and PiB PET findings for two cases of CAA-ri. We also provide a comprehensive literature review of the amyloid PET imaging features of previously reported cases of CAA-ri.

## Case presentation

### Case 1

An 88-year-old male presented with progressive changes to consciousness, lethargy, and gait disturbances for 2 months.

**Figure 1:**
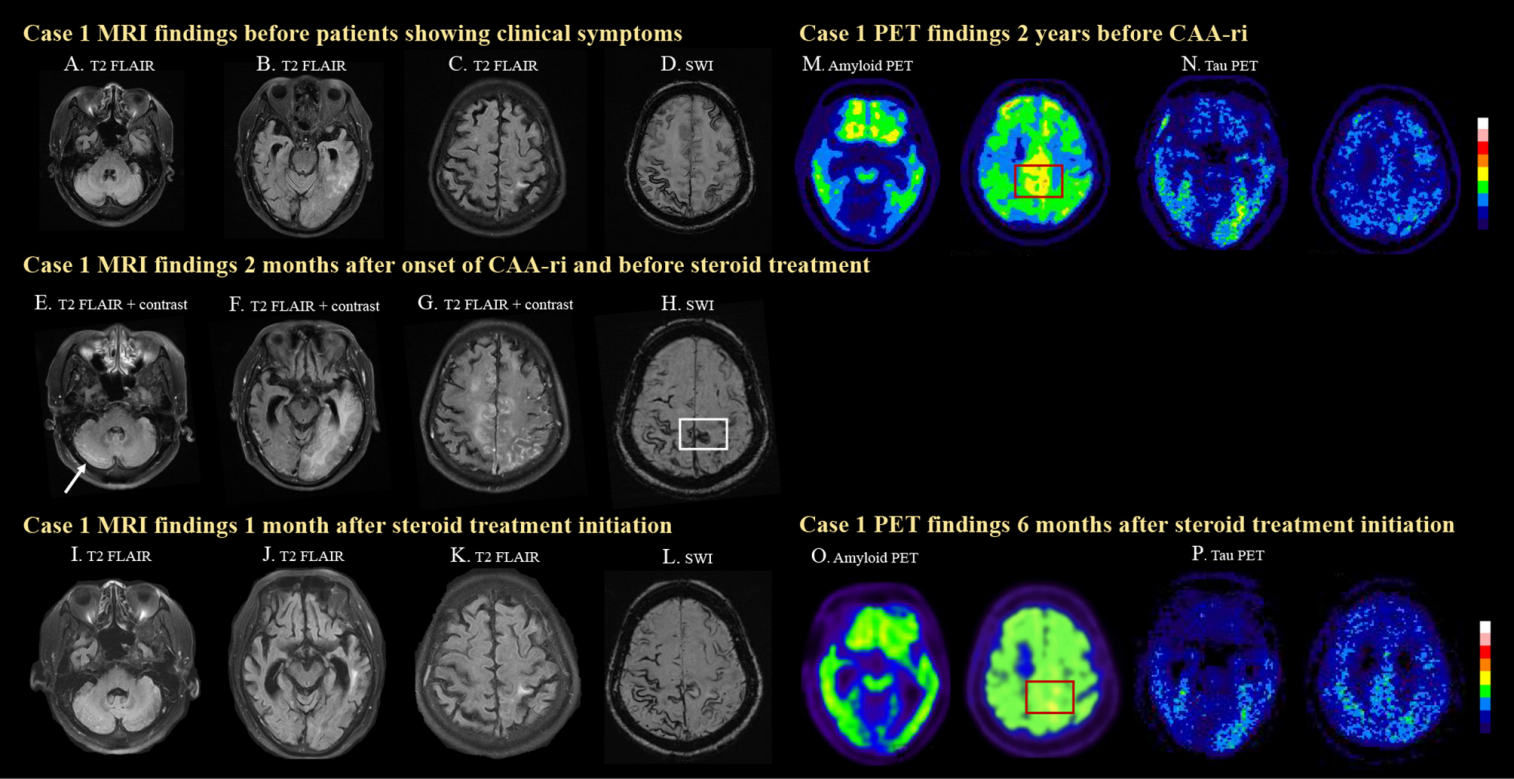
MRI and PET images for case 1. The case was diagnosed as probable CAA after a symptomatic left temporal ICH with disseminated cortical superficial siderosis (cSS) on MRI **(A–D)**. Two months prior to his clinical symptoms, mild left temporal T2 FLAIR hyperintensity was already seen **(B)**, which may suggest insidious onset of ARIA-E. The patient then developed progressive lethargy and spontaneous ARIA-E, showing white matter hyperintense lesions with leptomeningeal enhancement and mass effects in the right cerebellum [**(E)**, arrow], left temporal **(F)**, bilateral medial frontal, and parietal lobes **(G)** on T2 FLAIR images before steroid treatment. A newly developed cSS in the posterior medial frontal region [**(H)**, white box] was observed on SWI, which was not seen 2 months earlier **(D)**. The cerebral edema significantly improved after steroid treatment **(I–K)**. Progressively disseminated cSS was also observed **(L)**. High amyloid deposition in the posterior medial frontal region was detected on amyloid PET 2 years before CAA-ri [**(M)**, red box]. Amyloid deposition decreased in the medial frontal region 6 months after CAA-ri [**(N)**, red box]. The patient showed positive tau pathology on tau PET scans **(O, P)**.

The patient had a lobar intracerebral hemorrhage (ICH) in the left temporal lobe 10 years ago. The brain MRI showed multifocal cortical superficial siderosis (cSS) on susceptibility-weighted imaging (SWI) at that time. He was then diagnosed with probable CAA. The patient had returned to work after the ICH event but developed insidious short-term memory decline for 5 years. Significant decline in cognitive function was found, as the patient's Mini-Mental State Examination score decreased from 7 to 2 points in 1 year. Because of prominent cognitive decline, and diffuse deposition of amyloid beta especially in the bilateral fronto-parietal region on amyloid PET, concomitant Alzheimer disease (AD) was suspected clinically. The patient received a routine MRI follow-up which showed mild left temporal T2 FLAIR hyperintensity lesions (Figures 1A–D) and the patient was nearly as his usual condition at that time. Progressive lethargy, an unsteady gait, and dull response to stimuli were noted in the following 2 months.

Due to the progressive disturbances to consciousness, the patient was admitted for diagnostic work-up and management. Brain MRI revealed a leptomeningeal enhancement (Figures 1E–G) with edema of the bilateral high posterior frontal lobes, right medial parietal lobe, left temporo-parieto-occipital lobes and right cerebellum (Figure 1E), as well as new focal cSS in the posterior medial frontal region (Figure 1H, white box) that were absent in the MRI performed 2 months earlier (Figure 1D).

Cerebrospinal fluid (CSF) analysis showed elevated total protein (170.6 mg/dL) and the IgG index was 0.79. No other infection or malignancy was identified. An electroencephalogram (EEG) showed nearly continuous diffuse or generalized slow waves without responses to alerting maneuvers. The *APOE* genotype was ε2/ε3. Thus, the diagnosis of probable CAA-ri was made. Intravenous methylprednisolone (500 mg per day) was administered for 5 days. The patient's cognitive function improved 1 week after steroid initiation and lethargy reduced. Oral prednisolone (40 mg per day) was prescribed on a tapering course for 2 months.

Follow-up brain MRI 1 month later showed resolution of the edematous changes (Figures 1I–K). PiB PET performed 2 years earlier (prior to CAA-ri) showed diffuse deposition of amyloid beta especially in the bilateral fronto-parietal region, which may be the characteristic pattern of AD. However, higher amyloid deposition was noted in the posterior medial frontal regions (Figure 1F, red box), which overlapped with the location of the newly developed cSS (Figure 1H, white box). Amyloid PET was repeated 6 months after CAA-ri and revealed focally decreased amyloid uptake in the posterior medial frontal lobe (Figures 1M, O, red boxes) and the left temporal lobe. The regional cortical-to-cerebellum standardized uptake value ratio decreased from 1.73 to 1.67 in the left frontal area, from 1.76 to 1.63 in the right frontal area and from 1.46 to 1.30 in the left temporal area. The patient also underwent cerebral tau PET using 18F-T807 2 years earlier and 2 months after CAA-ri, which confirmed a cerebral phosphorylated-tau pathology (Figures 1N, P). The tau deposition appeared more correlated with region of CAA-ri than the amyloid deposition in the left temporal region.

During follow-up, the patient suffered new cerebral hemorrhages in the right frontal lobe and the left basal ganglion 1 year later and had a poor prognosis. He subsequently became functionally dependent.

### Case 2

A 72-year-old male patient had gradual short-term memory decline and word-finding difficulty for 2 years. However, his ambulation and instrumental activities of daily living were otherwise intact. He was ever admitted to our hospital because of systemic cryptococcosis and *Pneumocystis jirovecii* pneumonia infection. At that time, CSF study revealed elevated total protein (92.9 mg/dL); glucose and the lymphocyte count were within normal limits. India ink and cryptococcal antigen staining of CSF were negative. Brain MRI showed numerous infarcts and microbleeds in the bilateral cerebral and cerebellar hemispheres. During hospitalization, an acute change in consciousness to a comatose state occurred. Brain computed tomography (CT) revealed cerebellar hemorrhage with hydrocephalus. Therefore, the patient received suboccipital craniectomy and evacuation of the cerebellar hematoma. He recovered consciousness after surgery. He then resumed orientation and ambulation after amphotericin B treatment for systemic cryptococcosis and rehabilitation for 1 month. The patient was discharged with oral fluconazole and no events were observed during follow-up.

**Figure 2:**
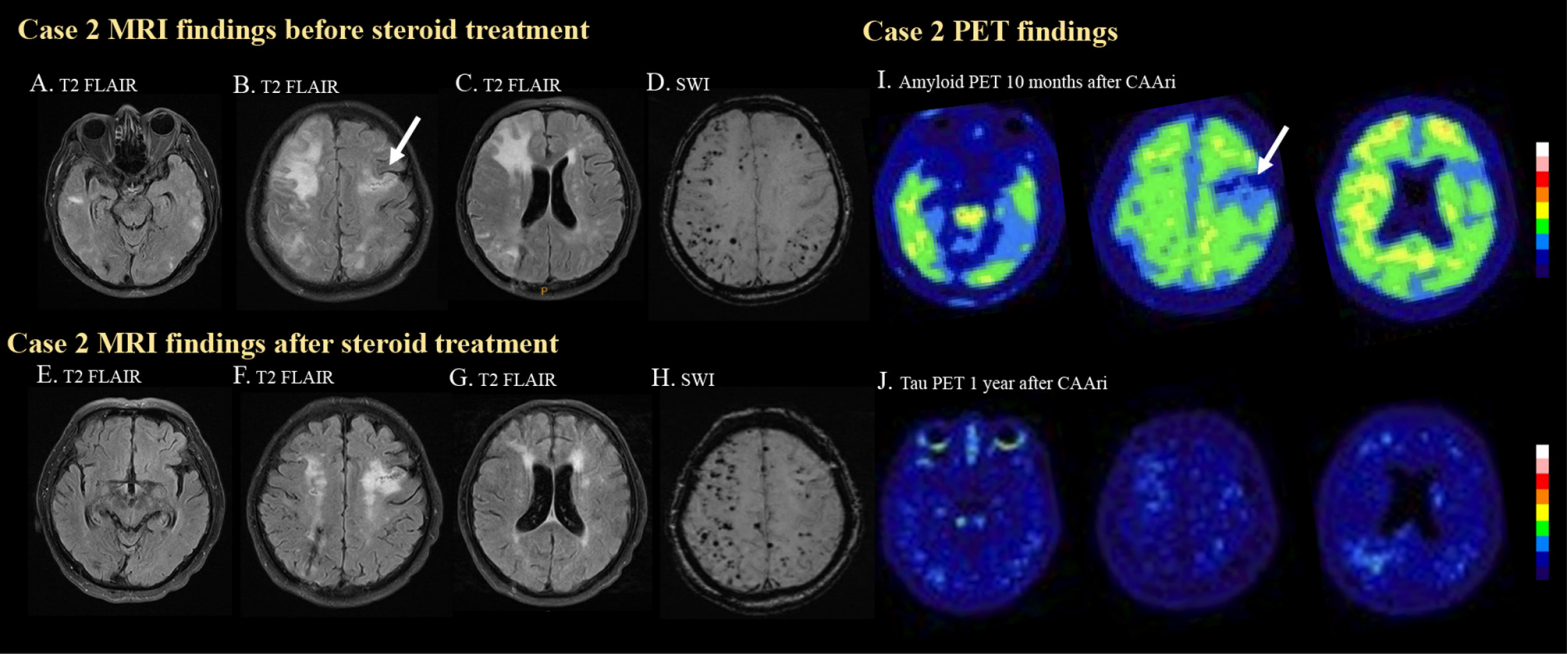
MRI and PET images for case 2. In the second case, multiple hyperintense foci in the bilateral temporal lobes **(A)** and cortical and subcortical edema in the right frontal and bilateral parietal regions **(B, C)** were observed on T2 FLAIR imaging before steroid treatment. Previous ischemic insult was also demonstrated [**(B)**, arrow]. Multiple strictly lobar microbleeds were detected in the bilateral cerebral hemispheres on SWI. **(D)** Follow-up T2 FLAIR imaging after steroid treatment showed resolution of the aforementioned edematous changes and hyperintense foci **(E–G)**. The microbleed became more prominent after the CAA-ri **(H)**. Amyloid PET confirmed amyloid deposition at 10 months after CAA-ri, but the uptake pattern did not correlate with the regions of FLAIR hyperintensities **(I)**. Tracer binding was not observed in the regions affected by previous ischemic insults [**(I)**, arrow]. No significant tau deposition was observed on a tau PET scan **(J)**.

However, 2 months after the discharge, he came to our hospital again due to newly developed symptoms including forgetfulness, ideational apraxia, left hemineglect, personality changes, and agitation for 3 weeks. He was then admitted for further survey. The brain MRI showed bilateral cerebral hemispheres FLAIR hyperintensities (Figures 2A–C) and multiple cortical-subcortical microbleeds (Figure 2D). Moreover, superficial siderosis were seen in the surface of brain stem and cerebellum (not shown in figure), which was probably owing to previous surgery of cerebellar hematoma evacuation. CSF study showed elevated total protein, but no evidence of cryptococcal infection or bacterial meningoencephalitis. EEG showed mild to moderate diffuse cortical dysfunction with emphasis in the right frontocentral area. Due to the patient's previous history, central nervous system (CNS) cryptococcosis was suspected. Intravenous induction therapy with amphotericin B and mannitol were initiated in the beginning, but the clinical symptoms of cognitive deficits persisted.

Due to the suspicion of probable CAA-ri, intravenous methylprednisolone (40 mg per day) was administered for 2 weeks. The cognitive function of the patient improved 1 week after the initiation of steroid therapy. Oral prednisolone treatment with a tapering course was prescribed for 2 months after discharge. Follow-up EEG normalized. Brain MRI (Figures 2E–G) 4 months after discharge showed resolution of the edematous changes, but the number of cerebral microbleed increased (Figure 2H).

The patient received amyloid PET about 6 months after the resolution of the edematous changes on MRI. The amyloid PET showed diffuse amyloid deposition especially in the bilateral frontal and posterior temporal regions. The higher amyloid burden area did not significantly correlate with the previous area of FLAIR hyperintensities. No significant tau deposition was noted (Figure 2J). His *APOE* genotype was ε3/ε3. His clinical status remained stable during outpatient follow-up.

## Discussion

We have presented two cases of CAA-ri with detailed clinico-radiological correlations, including the amyloid and tau imaging features. In the first case, the patient, who had previous symptomatic lobar ICH, received PET scans prior and after CAA-ri; these images revealed a focally decreased amyloid load in the posterior medial frontal lobe which corresponded to the region of amyloid related imaging abnormalities-edema (ARIA-E). Positive tau pathology was also noted. In the second case, the patient was initially suspected to have CNS cryptococcosis, but the diagnosis of CAA-ri was subsequently made due to the characteristic MRI features and good response to corticosteroid treatment. The amyloid scan showed positive amyloid deposition. However, neither case suggested a regional association between ARIA-E and higher amyloid uptake on PET before or after CAA-ri. To our knowledge, our first case is the first case report of longitudinal changes on amyloid scans in CAA-ri. These are also the first case reports of CAA-ri with the results of tau scans.

CAA-ri is a rare immune-mediated disorder of the CNS in the setting of CAA. Imaging studies, especially blood-sensitive MRI using T2^*^-weighted or susceptibility-weighted imaging (SWI), are important diagnostic modalities for CAA (14). Brain MRI of our two cases showed multiple microbleeds or cSS strictly in cortical-subcortical areas. These findings are compatible with the clinical diagnosis of probable CAA (15). Although supporting pathology results are not available, probable CAA-ri is diagnosed according to the clinical presentation, findings of CSF studies, and FLAIR MRI features of ARIA-E (6). Additionally, infectious, autoimmune, or paraneoplastic encephalitis, brain neoplasms, and other causes of rapidly progressive dementia should be considered as differential diagnoses in clinical practice. Notably, there is leptomeningeal enhancement extending to the right cerebellar cortex in the first case, which was also responsive to the steroid treatment. Infratentorial involvement of CAA-ri has been rarely reported in previous literature. Previous studies have demonstrated a close relationship between superficial cerebellar hemorrhagic lesions and CAA (16, 17), and thus we suspected the cerebellar involvement in our first case was related to CAA-ri as well.

Developments in amyloid imaging have provided a better understanding of the accumulation of beta-amyloid (Aβ) in the brain and may provide diagnostic evidence of CAA (10). In consideration of the limited reports of a clinical correlation between amyloid imaging and CAA-ri, we searched the PubMed database for all articles published in English that include the term: “cerebral amyloid angiopathy-related inflammation” plus at least one of the following terms: “amyloid imaging”, “amyloid PET”, “Pittsburgh compound B”, “florbetapir”, “flutemetamol”, and “florbetaben”. We selected case reports or case series of patients with probable CAA and evidence of recent inflammation with amyloid imaging published between 2014 and 2021. A total of five eligible studies (11–13, 18, 19) were retrieved (Table 1).

**Table 1:** Literature review of case reports and case series of CAA-ri with available amyloid PET scans.

**References**	**Number of CAA-ri cases with amyloid PET**	**Age, gender, and underlying disease**	**Clinical symptoms**	**Brain MRI characteristics**	**Type of amyloid PET and features**
Renpei Sengoku et al., (18)	2	72-year-old male with AD, neuropathological examination revealed severe amyloid deposits in the cortical and leptomeningeal blood vessel walls	Wandering behavior for 1 month, with acute progressive dementia and spatial disorientation	FLAIR High intensity area in right temporo-parieto-occipital white matter, with edema T2^*^WI Microbleeds, predominantly in dorsal regions of bilateral temporo-parieto-occipital lobes	11C-Pittsburgh Compound B Accumulated in the fronto-parieto-temporal lobe (no detailed description of when amyloid PET was performed)
		72-year-old male, receiving hemodialysis for membranous nephropathy	Sensory aphasia	FLAIR High intensity area in left temporal cortex SWI Subarachnoid hemosiderosis and microbleeds in left temporal cortex	11C-Pittsburgh Compound B (10 months after onset without immunosuppressive treatment, but symptoms had resolved) focal uptake in the left temporal lobe
Jacopo C. DiFrancesco et al., (11)	One patient with recurrent CAA-ri	69-year-old male, first event at age 62	Bilateral upper limb tremor for several months; no other clinical signs	FLAIR Hyperintense lesion in left temporal area SWI Appearance of new microbleeds in left temporal lobe	11C-Pittsburgh Compound B (Four months after remission) No differences in the area of the recent inflammatory lesion
Mar'ia Carmona-Iragui et al., (12)	2	73-year-old male	Acute left frontal syndrome and cognitive impairment	FLAIR Hyperintense white matter lesions (WML), predominantly in left frontal region T2^*^GRE Multiple diffuse lobar/cortical microbleeds	18F-Florbetapir (19 months after corticosteroid treatment) Lower Florbetapir uptake at previous swollen region than in the homonym contralateral areas
		68-year-old female	Subacute aphasia and cognitive impairment in previous 2 months	FLAIR Hyperintense WML in left temporoparietal region T2^*^GRE Lobar/cortical microbleeds, predominantly in left temporoparietal region	18F-Florbetapir (13 months after corticosteroid treatment) Lower Florbetapir uptake at previous swollen region than in homonym contralateral areas
Dimitri Renard et al., (13)	9	Median age of 74-years-old (range: 62–83); five males, four females	Not described in detail	FLAIR Hyperintense lesions SWI Very high numbers of CMBs (median: 769 per patient) in patients with CAA-ri and positive correlation between CMBs and hyperintense regions	Florbetaben Median of 43 days after MRI (Two patients had active CAA-ri, 7 patients had CAA-ri in remission phase) Negative correlation between hyperintense lesions and amyloid burden (statistically non-significant)
Kosuke Matsuzono et al., (19)	1	80-year-old male, with relapsing polychondritis (RP)	Headaches, hallucinations, cognitive disturbance 5 months after RP onset	FLAIR Hyperintensity in posterior region T2^*^WI Multiple microbleeds, asymmetrically distributed in bilateral posterior, temporal, and parietal lobes	11C-Pittsburgh Compound B (12 months after onset) Amyloid deposition in bilateral frontal lobes, lack of PiB uptake at areas of inflammation
Our case series	2	88-year-old male, with chronic stage 3 kidney disease and hyperlipidemia	Progressive consciousness and gait disturbances for 2 months	FLAIR Leptomeningeal enhancement and edema in the right cerebellum, bilateral high posterior frontal lobes, right medial parietal lobe, and left temporo-parieto-occipital lobe SWI Disseminated cortical superficial siderosis, with new lesions in bilateral high posterior fronto-parietal regions	11C-Pittsburgh Compound B (2 years before CAA-ri) Higher amyloid deposition in posterior medial frontal region (6 months after CAA-ri) Focally decreased amyloid deposition in posterior medial frontal region compared to prior scan
		73-year-old male, history of disseminated cryptococcosis	Executive function and memory decline with gait disturbances for 3 weeks	FLAIR Bilateral cerebral hemispheres FLAIR hyperintensities, mostly in right cerebrum. SWI (1) Multiple cortical-subcortical microbleeds, (2) Cortical superficial siderosis at brain stem surface, and cerebellum (probably owing to previous surgery of cerebellar hematoma evacuation)	11C-Pittsburgh Compound B (10 months after CAA-ri) Diffuse amyloid deposition especially in the bilateral frontal and posterior temporal regions

CAA-ri, cerebral amyloid angiopathy-related inflammation; PET, positron emission tomography; AD, Alzheimer's disease; MRI, magnetic resonance imaging; FLAIR, fluid attenuated inversion recovery; T2^*^GRE, T2^*^ gradient recalled echo; SWI, susceptibility weighted imaging; PiB, 11C-Pittsburgh Compound B; WML, white matter lesions; CMB, cerebral microbleeds; RP, relapsing polychondritis.

**Figure 3:**
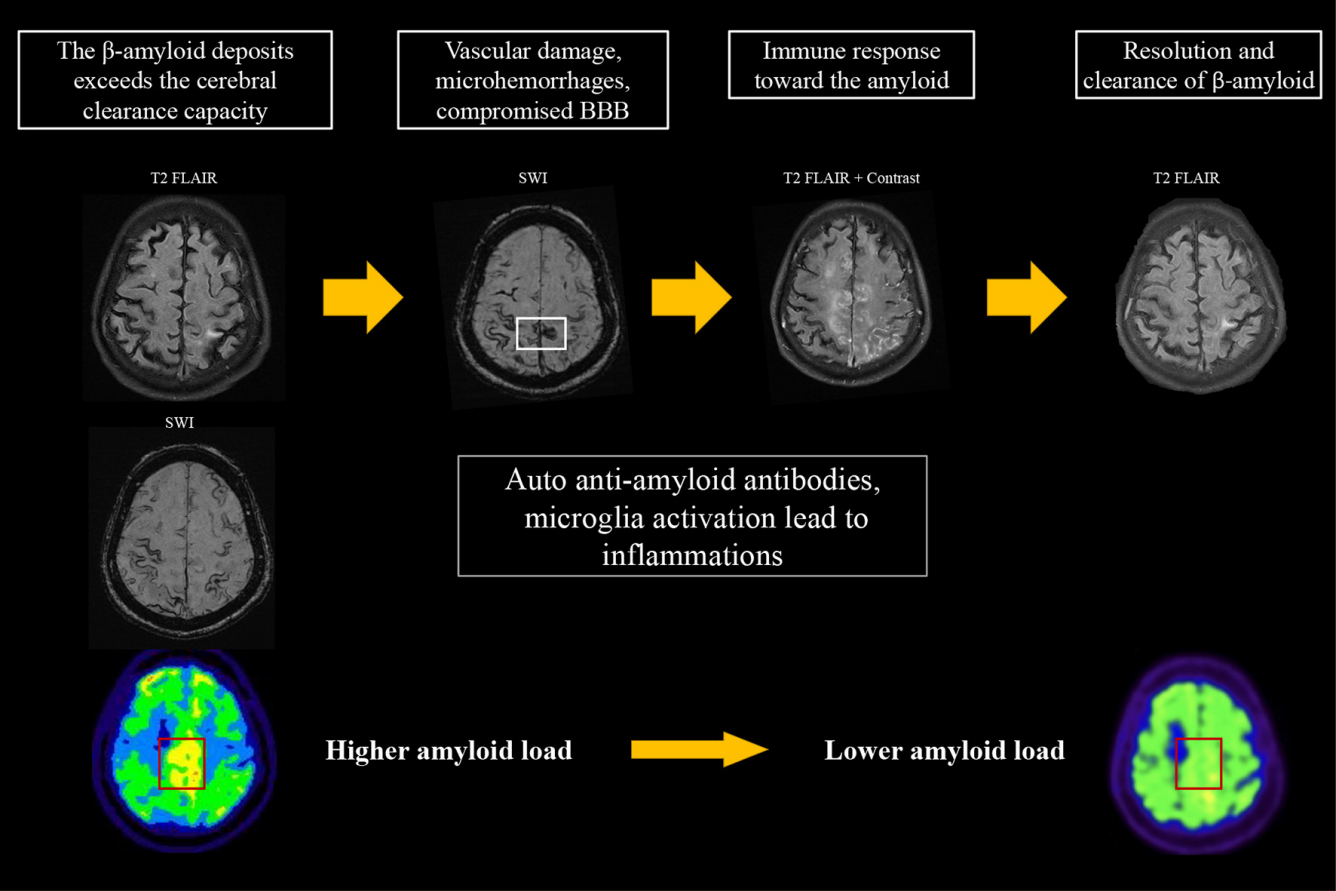
Proposed hypothesis for the changes on amyloid PET in CAA-ri. An increase in amyloid burden exceeds the clearance capacity, which may lead to focal vascular damage and microhemorrhages. This process induces an immune response that involves auto-antibodies and activation of microglia/macrophages for the clearance of β*-*amyloid, resulting in the typical MRI features of CAA-ri. The focal amyloid load appears lower when inflammation resolves.

According to the case reports published to date, a varied amyloid burden in the post-inflammatory regions is noted on PET. Sengoku et al. (18) found an increased focal amyloid burden at post-inflammatory lesions. DiFrancesco et al. (11) reported no difference in amyloid deposition in the area of recent inflammation. On the other hand, Carmona-Iragui et al. (12), Renard et al. (13), and Matsuzono et al. (19) described decreases in the amyloid burden in post-inflammatory areas; these authors hypothesized that CAA-ri may reflect the inflammatory process of amyloid clearance. These studies analyzed cross-sectional amyloid deposition, but did not assess the longitudinal changes in amyloid load. Their findings may suggest that although the amyloid burden is higher in post-ARIA-E regions compared to other areas of the brain, the true level of amyloid deposition in the ARIA-E regions may be lower than before inflammation. Our first case is the first report of CAA-ri for which amyloid scans were available before and after inflammation. At the time of CAA-ri, new cSS, edematous changes, and contrast enhancement were observed around the regions of high amyloid deposition detected on amyloid PET two years prior to the event. Interestingly, the amyloid burden in the posterior medial frontal area appeared to be lower than before the event, which is compatible with the previous hypothesis that regional amyloid is cleared via an inflammatory process (Figure 3). Based on our findings and previous studies, we hypothesize that a high focal amyloid burden leads to vascular damage and microhemorrhage, which triggers an immune response and local inflammation. Auto anti-amyloid antibodies and activation of microglia/macrophages may participate in this immune response (3–5, 12, 20, 21); our proposed mechanism is illustrated in Figure 3.

Tau pathology, one of the signature markers in AD, is frequently found in CAA. In our first case, tau deposition in the left temporal region seemed to associate with the area of CAA-ri. Tau aggregation may be related to astrocytic responses associated with vascular amyloidosis and vascular damage (4, 22). The deposition of tau could also be a concomitant pathology of AD as the tau deposition was most significant in the temporal region. We could not confirm the relationship between tau pathology and CAA-ri, since there was no obvious tau deposition in our second case. Whether co-pathology of AD and CAA influence the risk of CAA-ri remains unclear, and this knowledge may provide a different perspective in understanding the ARIA events in immunization clinical trials for AD. Notably, recent works by Piazza and others demonstrated a strong regional association between activated microglia and ARIA-E as well as CSF auto-antibodies levels (4, 23, 24). Higher microglial responses were also found in patients with concomitant CAA and AD than those without coexisting AD pathology (4). Thus, amyloid and tau imaging studies of other cases of CAA-ri are needed to further elucidate the roles of amyloid and tau deposition in the pathogenesis of CAA-ri.

The case reports have several limitations. First, there were no pathology findings to confirm the definite CAA-ri of the patients. Second, only one of our reported cases had longitudinal imaging follow-up of amyloid PET to demonstrate a reduction of amyloid load in the region with ARIA-E. More cases are needed to support this observation. Lastly, anti-amyloid autoantibody in CSF was not tested so the role of this autoantibody was uncertain in this case series.

## Conclusion

Our case reports highlight several points. First, CAA-ri could be considered in elderly patients who exhibit subacute cognitive decline and asymmetrical edema on brain imaging. Amyloid imaging has a potential to assist the diagnosis of CAA-ri, especially when other diagnoses cannot be ruled out. Only limited data is available on the longitudinal changes on amyloid PET. Our report is the first to show the temporal changes of amyloid PET in CAA-ri. Further analysis of additional cases may provide important insight into understanding the pathophysiology of CAA-ri.

## Data availability statement

The raw data supporting the conclusions of this article will be made available by the authors, without undue reservation.

## Ethics statement

The studies involving human participants were reviewed and approved by Institutional Review Board of National Taiwan University Hospital. The patients/participants provided their written informed consent to participate in this study. Written informed consent was obtained from the participants for the publication of this case report.

## Author contributions

J-YY and Y-TC drafted the manuscript. H-HT revised the manuscript. J-SJ provided critical revision and supervision. All authors approved the final version submitted for publication.
